# High saturation magnetization of *γ*-Fe_2_O_3_ nano-particles by a facile one-step synthesis approach

**DOI:** 10.1038/srep32360

**Published:** 2016-09-01

**Authors:** Derang Cao, Hao Li, Lining Pan, Jianan Li, Xicheng Wang, Panpan Jing, Xiaohong Cheng, Wenjie Wang, Jianbo Wang, Qingfang Liu

**Affiliations:** 1 Key Laboratory for Magnetism and Magnetic Materials of the Ministry of Education, Lanzhou University, Lanzhou 730000, People’s Republic of China; 2 Key Laboratory of Special Function Materials and Structure Design, Ministry of Education, Lanzhou University, Lanzhou 730000, People’s Republic of China; 3 Key Laboratory of nonferrous metals chemistry and resources utilization, Lanzhou University, Lanzhou 730000, People’s Republic of China

## Abstract

We have demonstrated the synthesis of *γ*-Fe_2_O_3_ nano-particles through a facile and novel calcination process in the air. There is no *p*H regulation, gas atmosphere, additive, centrifugation or other complicated procedures during the preparing process. A detailed formation process of the nano-particles is proposed, and DMF as a polar solvent may slower the reaction process of calcination. The structures, morphologies, and magnetic properties of *γ*-Fe_2_O_3_ nano-particles were investigated systematically, and the pure *γ*-Fe_2_O_3_ nano-particles obtained at 200 °C display uniform morphology good magnetic property. The saturation magnetization of obtained pure *γ*-Fe_2_O_3_ is about 74 emu/g, which is comparable with bulk material (76 emu/g) and larger than other results. In addition, the photocatalytic activity for degradation of methylene blue is also studied, which shows proper photocatalytic activity.

Magnetic nanomaterials have attracted much interest gradually, since these materials have many potential applications such as information storage, color imaging, magnetic refrigeration, gas sensors, ferrofluids, and photocatalysis etc.^1^,^2^,^3^,^4^,^5^,^6^,^7^ Recently, researches of magnetic nanomaterials are fascinating due to its powerful usefulness for a variety of biomedical^8^,^9^ and chemical engineering applications^10^. Among the magnetic nanomaterials, maghemite (*γ*-Fe_2_O_3_) is considered as one of the most desirable materials for various applications due to its inherent biocompatible nature and stability of oxidation as well as its good magnetic properties^1^,^11^,^12^,^13^. *γ*-Fe_2_O_3_ also exhibits modest photocatalytic activity and separability^7^,^14^. It can be used associated with ZnO or TiO_2_ to enhance the visible light adsorption and increase the electron/hole separation^15^,^16^. High magnetization of *γ*-Fe_2_O_3_ has potential applications for cleaning polluted water with the help of magnetic separation. As a result, magnetic properties, as an important symbolic characteristic of *γ*-Fe_2_O_3_ nano-particles, are noticeable for study.

Therefore, new approaches for the synthesis of *γ*-Fe_2_O_3_ particles as well as the investigation on their properties are of fundamental importance for the development of science and technology. The basic and conventional route of these methods or processes for the synthesis of *γ*-Fe_2_O_3_ nano-particles are controlling the oxidation of Fe_3_O_4_^17^,^18^,^19^,^20^, and the total preparation process is shown as follows:


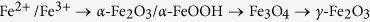


Various methods have been reported for the synthesis of *γ*-Fe_2_O_3_ nano-particles, such as coprecipitation^12^,^21^, hydrothermal^22^, microemulsions^4^,^23^, thermal decomposition^24^,^25^, aerosol pyrolysis^26^, sole-gel^27^, hydrosol chemical reaction^20^, combustion synthesis^7^, Massart method^2^,^28^, solvothermal method^29^,^30^, wet chemical method^31^,^32^, sonochemical route^33^, ultrasonic decomposition^34^, high-temperature solution reaction^1^, chemical reaction^6^, and other chemical process^35^,^36^,^37^,^38^,^39^. These synthesis processes or methods are the important routes for the synthesis of *γ*-Fe_2_O_3_ nano-particles and its composite materials. However, suitable *p*H value, long reaction time, and definite additives or surfactants are indispensable for obtaining pure*γ*-Fe_2_O_3_ nano-particles with controllable morphology during those synthesis processes. Furthermore, centrifugation and purification are the vital factor for nano-particles with single products and good dispersity. Those preparation processes were totally complicated and cumbersome. Significantly, the saturation magnetization of *γ*-Fe_2_O_3_ nano-particles of the most methods mentioned above is still dissatisfied.

Herein, we report a unified approach for the synthesis of *γ*-Fe_2_O_3_ nano-particles in the air via a facile and novel calcination process. The method is different from the earlier approaches, and there is no *p*H regulation, gas atmosphere, centrifugation and other supplementary reagents during the preparing process. Various characterizations were measured to perform the obtained pure *γ*-Fe_2_O_3_ nano-particles, and *γ*-Fe_2_O_3_ nano-particles show a high saturation magnetization. In addition, the photocatalytic activity of *γ*-Fe_2_O_3_ nano-particles was also studied.

## Methods

**Figure 1:**
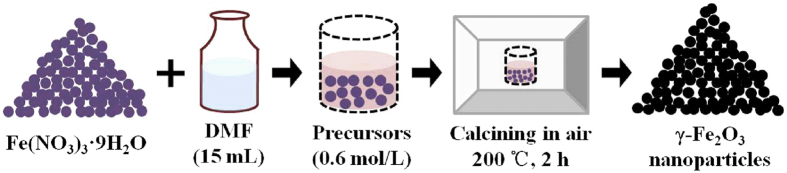
The schematic diagram of experimentation.

A unified method was provided using a simple and convenient route to assemble γ-Fe_2_O_3_ nano-particles. Ferric nitrate was dissolved in Dimethyl Formamide (DMF), the precursor was 0.6 mol/L, and calcined at different temperature (100 °C~400 °C, the interval is 50 °C) for 2 hours in the air. The heating rate was 1 °C/min. The schematic diagram of experiment is shown in Fig. 1.

The crystal structure of samples were measured by X-ray diffraction (XRD, PANalytical X’Pert) equipped with Cu-Kα radiation (λ = 1.5406 Å). The morphology of all samples was observed by using field emission scanning electron microscopy (FESEM, Hitachi S-4800) and transmission electron microscopy (TEM, Tecnai^TM^ G^2^ F30, FEI) equipped with an energy-dispersive spectrometer (EDS). The X-ray photoelectron spectroscopy (XPS, PHI-5702, Physical Electronics) were performed using a monochromatic Al-Kα irradiation and a charge neutralizer. All binding energies were referred to the C1 s peak at 284.6 eV of the surface adventitious carbon. The magnetic properties of the samples were measured by a vibrating sample magnetometer (VSM, Lakeshore 7304). The measurement process of surface areas and photocatalytic activity of the sample were shown in the Supporting Information (SI).

## Results and Discussion

**Figure 2:**
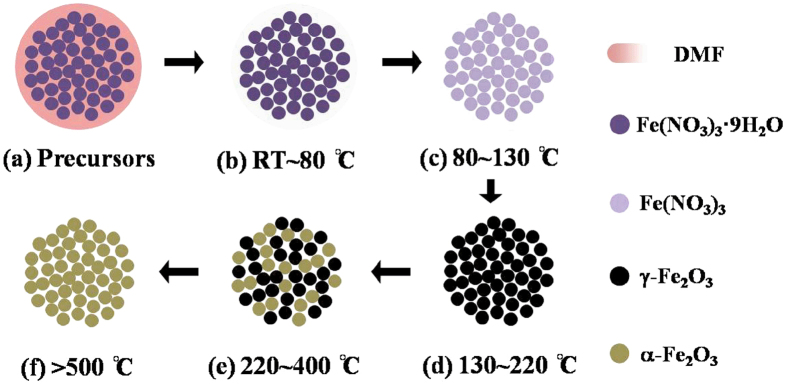
The formation mechanism of *γ*-Fe_2_O_3_ nano-particles.

On the basis of the below experiments and results, a formation mechanism of the nano-particles in this work is proposed, which is outlined in Fig. 2. It is suggested that the following reactions occur during the calcination process:


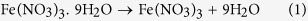



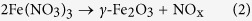



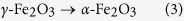


As shown in Fig. 2(a), the precursor is composed of DMF and iron nitrate. The solvents begin to volatilize at the beginning of the heating process (Fig. 2b). DMF in precursors plays a role of solvent, which helps the diffusion and contaction of the reactant molecules in the course of volatilization. When calcination temperature (CT) is increased (Fig. 2c), DMF is exhausted gradually, and the iron nitrite nonahydrate (Fe(NO_3_)_3_·9H_2_O) loses its water of hydration. The processes of Fig. 2(a–c) reveal that the surface morphology of sample seems not to be changed from their nature. The corresponding SEM picture and XRD pattern at 100 °C show bulk grains, suggesting an amorphous structure. The non-magnetic results (VSM loop) also confirm the amorphous structure. When the specimen is calcined at a moderate temperature (Fig. 2d), Fe(NO_3_)_3_ decomposes into *γ*-Fe_2_O_3_, and the nucleation process is observed (See SEM image and XRD spectrum at 150 °C). When CT then increases, a large area of *γ*-Fe_2_O_3_ nucleation is crystallized to the uniform nano-particles, which can be demonstrated by SEM image and XRD spectrum of 200 °C. Thereafter, when CT is about 220~400 °C (Fig. 2e), *γ*-Fe_2_O_3_ nano-particles begin to engulf its surrounded crystals, and various sizes of *α*-Fe_2_O_3_ particles are formed. This can be supported by SEM images and XRD spectra of 250~300 °C. As CT is further improved, more *γ*-Fe_2_O_3_ particles are transformed to *α*-Fe_2_O_3_, and the morphologies and structures of samples are mainly composed of *α*-Fe_2_O_3_ particles (see SEM images and XRD spectra of 350~400 °C). Finally, when CT exceeds 500 °C, all *γ*-Fe_2_O_3_ is disappeared, and plenty of *α*-Fe_2_O_3_ nano-particles are achieved. The result can be confirmed by the previous phase transformation studies^2^,^38^,^40^.

To sum up, combining all results of γ-Fe_2_O_3_ nano-granules, the detailed formation processes are discussed below. DMF is a commendable solvent when compared with water, and DMF is associated well with the cation^41^,^42^. As a solvent, DMF could disperse the ions, and coats each ion during the calcination process. As shown in Figure S1–S2 of SI, α-Fe_2_O_3_ particles are produced in the water but it cannot generate in DMF under the same experiment condition (1 °C/min, 200 °C). This indicates DMF may slower the reaction process of calcination, which restrains the transformation process of γ-Fe_2_O_3_ to α-Fe_2_O_3_. When CT or heating rates is increased, this restraint will be weakened.

**Figure 3:**
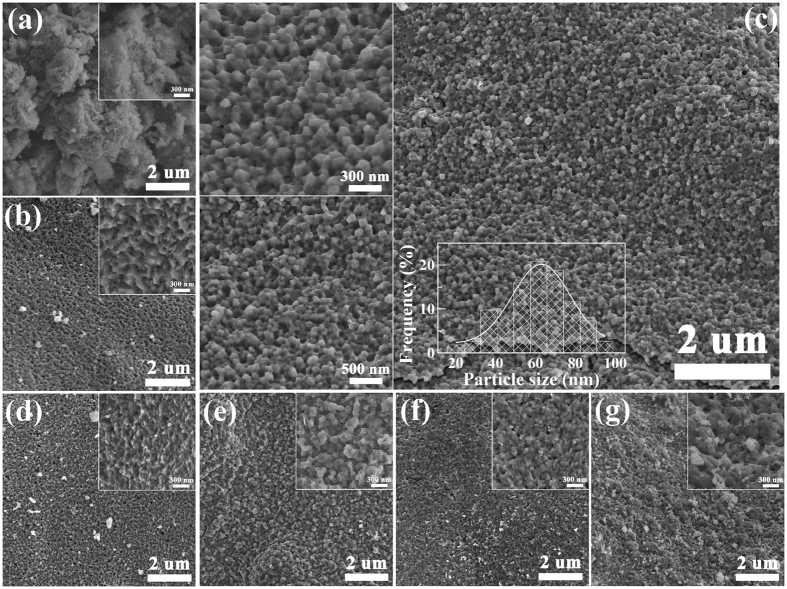
SEM images of of nano-particles of different calcinations temperature (**a**) 100 °C, (**b**) 150 °C, (**c**) 200 °C, (**d**) 250 °C, (**e**) 300 °C, (**f**) 350 °C, and (**g**) 400 °C, respectively. The inset of (**c**) is the size distributions of nano-particles at 200 °C.

Figure 3 shows SEM images of the samples of different CT. It can be seen that the morphologies change obviously with the increased CT. The sample of 100 °C (Fig. 3a) presents a number of disorderly bulk particles, and the shape of particles changes to compact and well-regulated nano-particles at 150 °C (Fig. 3b). Especially when CT is 200 °C (Fig. 3c), a large quantity of uniform nano-particles with the size about 60 nm are achieved. However, when CT is further improved (250~400 °C of Fig. 3d–g), the particles display an increased grain growth (Fig. 3d,e). The particle size becomes nonuniform, and strong piece of reunited particles are observed with the improvement of CT (Fig. 3f,g). These results are detailedly studied by the following techniques.

**Figure 4:**
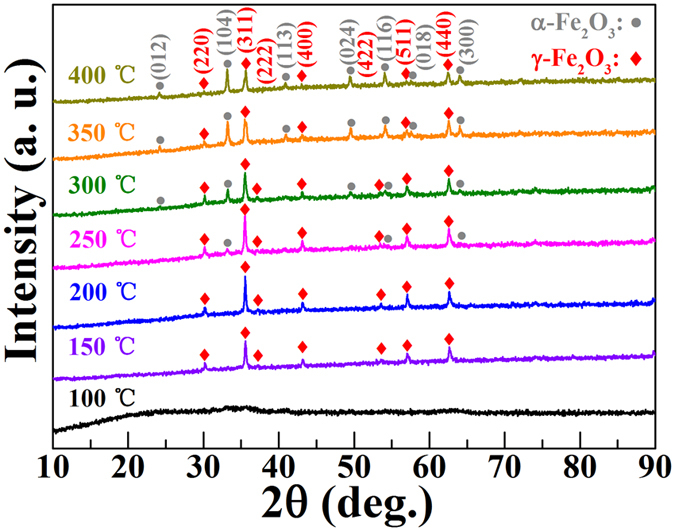
XRD patterns of *γ*-Fe_2_O_3_ nano-particles at different calcinations temperature.

XRD data were used to determine the structural parameters of all the samples. Figure 4 shows XRD patterns of all the samples at different CT. It can be observed that when CT is 100 °C, the sample is not crystallized due to the slow evaporation of DMF. Thus, there are no peaks could be seen in XRD pattern. Afterwards, the diffraction spectra of samples reveal the good single *γ*-Fe_2_O_3_ phases (150 °C and 200 °C) with the cubic crystal system (JCPDS#39-1346), and all the diffraction peaks can be well indexed. It can be confirmed that the particles are *γ*-Fe_2_O_3_ rather than *α*-Fe_2_O_3_, due to the different XRD pattern of *α*-Fe_2_O_3_ (JCPDS#80-2377) and *γ*-Fe_2_O_3_^17^,^19^. The average crystalline size estimated from Scherrer analysis is about 36 nm (150 °C) and 32 nm (200 °C) for γ-Fe_2_O_3_. In addition, the peak intensity of *γ*-Fe_2_O_3_ calcined at 200 °C is stronger than that of 150 °C, which indicates that the nano-particles present higher crystallization at 200 °C. However, the samples display *α*-Fe_2_O_3_ phases when CT exceeds to 250 °C. The emergence of additional impurity phase, i.e., *α*-Fe_2_O_3_, is more obvious with the enhanced temperature (300~400 °C). These XRD results indicate that the oxide of iron cannot be formed when CT is 100 °C. When CT is increased to 150~200 °C, *γ*-Fe_2_O_3_ nano-particles can be achieved, and *α*-form is observed as CT exceeds 250 °C. It is well-known that *γ*-Fe_2_O_3_ can be further transformed into *α*-Fe_2_O_3_ at higher temperature^33^,^40^, and the phase transformation temperature in our research (250 °C) is similar to the previous literature^2^,^40^,^43^.

**Figure 5:**
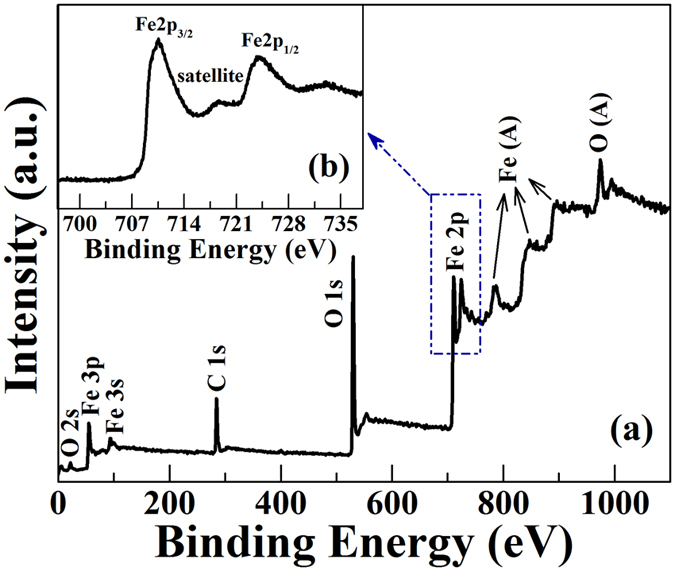
XPS patterns of *γ*-Fe_2_O_3_ nano-particles under 200 °C. (**a**) Full scanned XPS spectra, (**b**) XPS spectra of Fe 2p core-level.

In order to distinguish the chemical composition of *γ*-Fe_2_O_3_ as opposed to Fe_3_O_4_, XPS measurement of pure *γ*-Fe_2_O_3_ nano-particles is further performed, which is displayed in Fig. 5. The full scanned XPS spectra of *γ*-Fe_2_O_3_ sample of 200 °C in the range of 0–1200 eV were shown in Fig. 5(a). Except for Fe 2p, O 1s, and C 1s peaks in the spectra, no redundant peaks appear together in *γ*-Fe_2_O_3_ nano-particles, and C element belongs to the carbon contaminants absorbed on the surface of the tested samples. In particular, Fe 2p_3/2_ spectra (Fig. 5b) exhibit two peaks at 710.6 and 724.1 eV, which are the characteristic peaks of the 3+ ion of *γ*-Fe_2_O_3_^3^,^19^, and there is no signal or shoulder at smaller binding energies as would be expected for the presence of the Fe^2+^ ion (~708 eV)^44^,^45^. Furthermore, an additional peak at about 718.7 eV is the shakeup satellite peak, which also indicates the absence of the Fe^2+^ ion^3^,^44^. XPS results are consistent with the judgment of XRD spectra.

**Figure 6:**
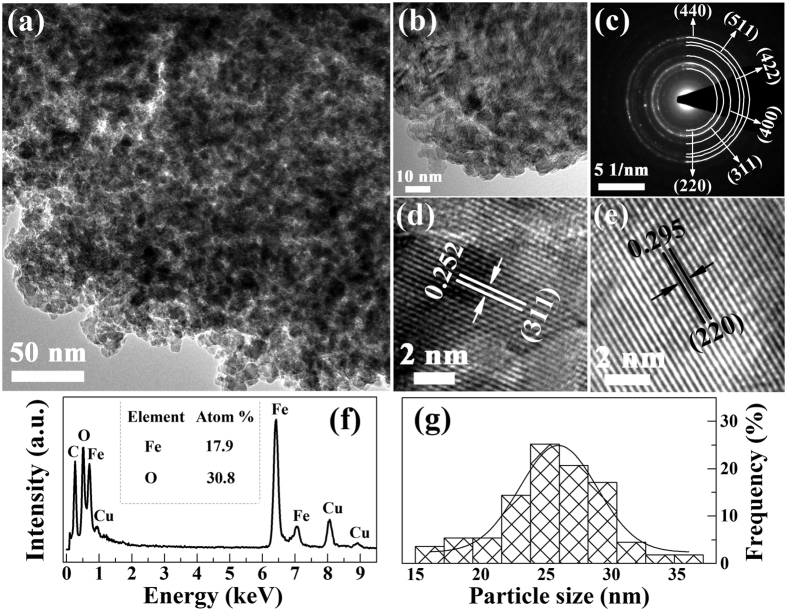
Typical TEM images (**a**,**b**), SAED (**c**), HRTEM image (**d**,**e**), EDX (**f**), and grain size distributions of *γ*-Fe_2_O_3_ nano-particles of 200 °C.

As a representative, the morphology and structure of the pure *γ*-Fe_2_O_3_ nano-particles (200 °C) are further characterized by TEM. As shown in the Fig. 6(a,b), the results indicate large and black areas of near-spherical *γ*-Fe_2_O_3_ nano-particles, and the granules present a low dispersity, which may be due to the reuniting of the nano-particles. HRTEM characterizations show the lattice fringes of the obtained ferrites, and the interfringe distance shown in Fig. 6(d,e) are 0.252 nm and 0.295 nm, which are correspond well to {311} and {220} planar spaces of *γ*-Fe_2_O_3_ nano-particles, respectively. Both the lattice fringes and SAED (Fig. 6c) clearly presents a group of atomic planes within each particle, revealing the highly crystalline nature of these nano-particles. Meanwhile, Fig. 6(f) gives EDX data of *γ*-Fe_2_O_3_ nano-particles, and the appearance of Cu peaks results from copper net used in the experiment. The element ratio of Fe:O is calculated to be 17.9:30.8, which is very close to the stoichiometry of *γ*-Fe_2_O_3_, which further confirms that the composition and structure are coincident with the chemical formulation of *γ*-Fe_2_O_3_. The particle size distributions, obtained from TEM micrographs, are shown in Fig. 6(g), and the histograms show that the samples display uniform particle distributions. The mean particle sizes obtained from the Gaussian fit of the histograms are 27 ± 2 nm, which is comparable with XRD line width results. However, size distributions obtained from SEM are larger than that of TEM, which may be due to the superimposed crystal or the compact arrangement of particles, and the shadow or astigmatism of the nano-particles could also cause the measurement error^46^. Some reports^47^ also show a relatively large particle size distribution.

**Figure 7:**
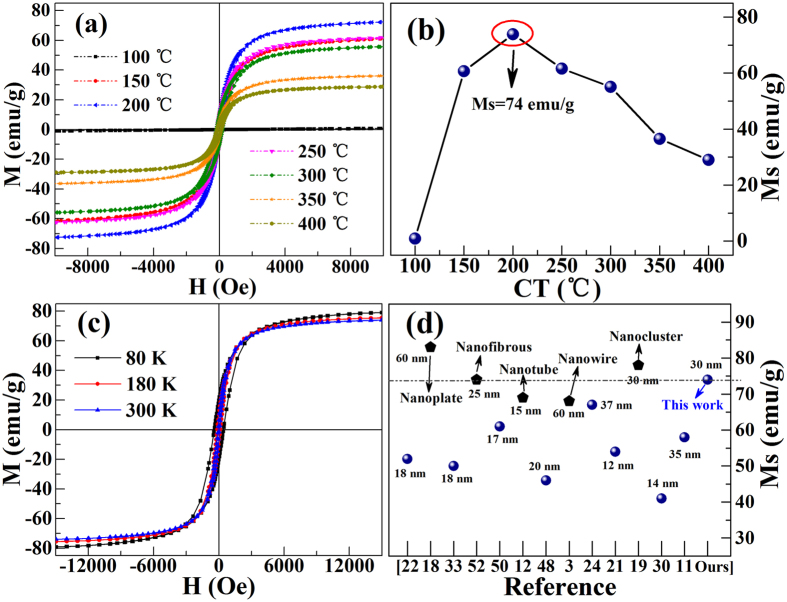
(**a**,**b**) Room temperature *M-H* loops for *γ*-Fe_2_O_3_ nano-particles and its corresponding *M*_s_ of different CT; (**c**) Temperature dependence of hysteresis loops measured at 80 K, 180 K, and 300 K of the pure *γ*-Fe_2_O_3_ nano-particles of 200 °C. (**d**) Comparisons of our work and other typical results of the particles size and *M*_s_ of *γ*-Fe_2_O_3_ nano-particles. The samples have been marked with circle (nano-particles) and pentagon (others), the values of the size represents the diameter of the samples.

On the base of good understanding of the microstructure and chemical phase of nano-particles, the room temperature magnetic performance of products is discussed below. As shown in Fig. 7(a), the non-crystalline sample (100 °C) has no magnetism. When CT is 150 °C, i.e. *γ*-Fe_2_O_3_ appears, the nano-particles emerge strong magnetism immediately, and the saturation magnetization (*M*_s_) is about 61 emu/g. Particularly, when CT reaches to 200 °C, *M*_s_ increases to 74 emu/g, which is comparable with bulk *γ*-Fe_2_O_3_ sample (*M*_s_ = 76 emu/g)^34^,^48^,^49^ but larger than other *γ*-Fe_2_O_3_ nanoparticles^11^,^30^,^33^,^50^. The higher values of *M*_s_ are due to the better crystals of the nanoparticles, consistent with both the XRD and HRTEM data. A recent work also shows that the higher crystallinity is benefited to enhanced *M*_s_ of the sample^51^. Typical comparative results of variable quantity of *M*_s_ are shown in Fig. 7(d). It can be seen *M*_s_ of this work is higher than other nanoparticles, but less when compared with nanoplate and nanocluster. However, when CT exceeds 250 °C, *M*_s_ is decreased monotonously. That is because that the presence of non-magnetic α-Fe_2_O_3_ leads to the relative reduction of magnetic *γ*-Fe_2_O_3_, and the magnetic moments total quality drop. When CT is further increased to 250~400 °C, the impurity α-Fe_2_O_3_ appears more obviously (which can be confirmed by XRD results), and *M*_s_ is reduced gradually. Furthermore, low temperature hysteresis loops of *γ*-Fe_2_O_3_ (200 °C) have also been carried out at 80 K, 180 K, and 300 K, which are shown in Fig. 7(c). The low temperature is realized by the liquid nitrogen. As expected, *M*_s_ and coercivity are also enhanced at low temperature. It is well-known that the coercivity and *M*_s_ will increase when the temperature decreases^34^,^50^.

The degradation of methylene blue (MB) was performed as a model reaction to investigate the photocatalytic activity of the sample, which was shown in Figure S3. The results reveal that *γ*-Fe_2_O_3_ has a little adsorption ability of MB with proper photocatalytic activity, which can degrade 16% MB dye in 60 min under UV irradiation. The photocatalytic activity of this work is comparable with the previous report^52^. As a result, the results provide the fabrication of Fe-based nanocomposites as proper performance photocatalysts, and high magnetization of *γ*-Fe_2_O_3_ also has potential in addressing environmental protection issues. The Brunauer-Emmett-Teller (BET) surface areas of *γ*-Fe_2_O_3_ nano-particles were measured to be 24.8 m^2^/g, which is smaller than the mesoporous nano-particles^24^,^29^,^30^. This suggests that the content of mesopores in the sample is considerably low. BJH average pore diameters calculated from the adsorption branch of the isotherms is 4.7 nm for the *γ*-Fe_2_O_3_ nano-particles, and the corresponding total pore volume is 0.04 cm^3^/g.

Various above investigations have demonstrated that a number of uniform and smooth *γ*-Fe_2_O_3_ nano-particles are obtained using calcination process in the air. Significantly, some typical methods or processes as the comparative results are discussed, and the comparative data are presented in Table 1. Except for the iron source of the preparing process, Table 1 detailedly shows the experiment parameters of solvent, additive or surfactant, *p*H, reaction time, centrifugation, and other parts of various methods. These methods or processes are not limited to the literatures we provided. As a result, although the dispersity and size of nano-particles in our research are not good with some of the previous reports, this technique only needs one solvent during the preparing process, and other additional processes are omitted. The mehtod realizes a simple, rapid and convenient route for assembling *γ*-Fe_2_O_3_ nano-particles when compared with others.

## Conclusions

We reported a unified approach for the synthesis of *γ*-Fe_2_O_3_ nano-particles via a facile and novel calcination process in the air. The process is no *p*H regulation, gas atmosphere, additive, centrifugation or other procedure during the experiment. The obtained pure *γ*-Fe_2_O_3_ nano-particles at 200 °C display good uniformity, and *α*-Fe_2_O_3_ will be observed when CT exceeds 250 °C. As a result, DMF is a commendable solvent when compared with water, which could well disperse the ions, and coats each ion during the calcination process. DMF may slower the reaction process of calcination, which restrains the transformation process of γ-Fe_2_O_3_ to α-Fe_2_O_3_. The saturation magnetization pure *γ*-Fe_2_O_3_ is about 74 emu/g, which is comparable with bulk material. In addition, the photocatalytic activity of the obtained nano-particles for the degradation of methylene blue shows proper photocatalytic properties.

## Additional Information

**How to cite this article**: Cao, D. *et al.* High saturation magnetization of *γ*-Fe_2_O_3_ nano-particles by a facile one-step synthesis approach. *Sci. Rep.* **6**, 32360; doi: 10.1038/srep32360 (2016).

## Supplementary Material

Supplementary Information

## Figures and Tables

**Table 1:** Compare the typical methods or processes with ours.

Method or process	Solvent or Additive or surfactant	*p*H	Main reaction conditions	Centrifugation or wash	Others	Particle size (nm)	*M*_s_ (emu/g)
solvothermal^30^	water, H_2_O_2_, D-(+)-C_6_H_12_O_6_, C_6_H_12_O_6_, oleic acid	/	400 °C, 0.5 h	ethanol	Need ultrasound, dried using H_2_O_2_	~12	26–42
wet chemical method^32^	HCl, water, TEOS ethanol, ammonia aqueou solution,	9.7	2 h, 30 min, 60 min	DMF, ethanol, water	using water and ethanol dried at 70 °C	17–29	/
high-temperature solution reaction^1^	diphenylether, 1,2-hexadecanediol, oleic acid, oleylamine	/	Ar flow all time, 200 °C 30 min, 265 °C 30 min	ethanol	Need Ar	6.4	/
sole-gel^27^	PVP, vinyl alcohol, saturated metal nitrate	/	150 °C, 2 h, 400 °C, 4 h	/	thermal degradation	30–50	/
microemulsions^23^	octyl ether, oleic acid, (CH_3_)_3_NO, ethanol	/	100 °C, 1 h, 130 °C, 2 h, reflux 1 h	ethanol	Need Ar	4–16	/
Massart’s method^28^	NaOH, HNO_3_, FeNO_3_	/	450~1200 °C, 30 min	acetone	/	4	/
thermal decomposition^24^	Ethanol, CTAB, CON_2_H_4_	/	2.5 h, 200 °C 1 h	ethanol	Dried at 45 °C	28–37	8–67
wet chemical method^32^	HCl, water, TEOS ethanol, ammonia aqueou solution,	9.7	2 h, 30 min, 60 min	DMF, ethanol, water	Dried at 70 °C using water and ethanol	17–29	/
coprecipitaion^12^	Water, ammonium hydroxide, urea, CTAB	10~11	2~3 h, 70 °C	water	Need vacuum, 120 °C	10	69.8
aerosol pyrolysis^26^	Water, oxalic acid, ammonia aqueou solution, KIO_3_	/	~300 °C, ~500 °C	water	Need nitrogen	50–120	/
combustion method^7^	glycine, ammonium nitrate, starch, polyethylene glycol	/	400 °C 2 h	/	Complex collection process	45–55	/
chemical reaction^6^	ethanol, water, hexane, 1-octadecene, oleic acid, sodium oleate	/	70 °C, 4 h	water, ethanol	Dried 320 °C, 0.5 h	5–22	/
sonochemical route^33^	Decahydronaphthalene, pentane	/	300 °C, 400 °C, 450 °C, 3 h	Yes	Ultrasonic 2 h, need vacuum	/	50
ultrasonic decomposition^34^	anhydrous decane, pentane	/	room temperature, sonicate for 3 h	/	Dried under vacuum 300 °C, 3 h	25	38–55
hydrothermal^22^	Water, MOE, acetylacetone,	Yes	140 °C, 4 h	acetone	Dried overnight under N_2_	12–26	53–73
hydrosol chemical reaction^20^	Water, HCl, NaOH	11~12	Papered Fe_3_O_4_ then Fe_3_O_4_ was oxidated for 30 min at about 100 °C	water, HCl	Complex reaction process	20–50	/
This work	DMF	No	200 °C, 2 h	No	No	~30	74

These methods or processes are not confined to the literatures we list.
