# Supplementary material for: High saturation magnetization of γ-Fe2O3 nano-particles by a facile one-step synthesis approach
Source: Sci Rep. 2016 Sep 1;6:32360. doi: 10.1038/srep32360 (PMC5007676; doi:10.1038/srep32360)
Supplement: Supplementary Information [file srep32360-s1.doc]

**Supporting Information**

**High saturation magnetization of *γ*-Fe_2_O_3_ nano-particles by a facile one-step synthesis approach**

Derang Cao^a^, Hao Li^b^, Lining Pan^a^, Jianan Li^a^, Xicheng Wang^b^, Panpan Jing^a^, Xiaohong Cheng^a^, Wenjie Wang^c^, Jianbo Wang^a,b^, Qingfang Liua[[1]](#footnote-2)*

^a^Key Laboratory for Magnetism and Magnetic Materials of the Ministry of Education, Lanzhou University, Lanzhou 730000, People’s Republic of China

^b^Key Laboratory of Special Function Materials and Structure Design, Ministry of Education, Lanzhou University, Lanzhou 730000, People’s Republic of China

^c^key laboratory of nonferrous metals chemistry and resources utilization, Lanzhou University, Lanzhou 730000, People’s Republic of China

**Experiment**

The sample was synthesized in the water at 200^o^C for 2 hours, and the heating rate was 1^o^C/min. The samples were also synthesized in DMF with different heating rates, which are 1^o^C/min, 2^o^C/min, 3^o^C/min, 4^o^C/min, and 5^o^C/min respectively. The calcination temperature is 200^o^C for 2 hours.

**Evaluation of surface areas and photocatalytic activity**

The surface area was measured by using the Brunauer-Emmett-Teller (BET) method (ASAP 2020, USA). The photocatalytic activity of the sample was evaluated by measuring the degradation ratio of methylene blue (MB). The initial concentration of MB solution was 5 mg L^-1^ and the used amount of photocatalyst was 0.1 g per 80 mL of MB solution. After the sample suspension was stirred for 40 min in the dark to realize the adsorption/desorption equilibrium, the photocatalytic reaction was started using a 500 W Hg lamp (0.337 mW/cm^2^) as the irradiation source. A series of a certain volume of suspension was withdrawn at selected times for analysis. After recovering the catalyst by centrifugation, the concentration of MB solution was analyzed by measuring the light absorption of the clear solution at 664 nm using a spectrophotometer (WFJ-7200, Unico, Franksville, WI, USA).

**Results and discussion**

XRD patterns of Figure S1 (a) indicate that the α-Fe_2_O_3_ phase is appeared when the heating rate is improved, and α-Fe_2_O_3_ phase becomes more obvious with the increased heating rate. The results reveal that the higher heating rate is helpful for the formation of α-Fe_2_O_3_. VSM loops of Figure S1 (b) also display a reduced *M*_s_ with the improvement of heating rate. This is because the presence of non-magnetic α-Fe_2_O_3_, which leads to the relative reduction of magnetic *γ*-Fe_2_O_3_, and the magnetic moment total quality drops. In addition, XRD results of sample synthesized in water shows a standard α-Fe_2_O_3_ phase, indicating γ-Fe_2_O_3_ phase cannot be formed in water under 200^o^C. VSM loop of sample synthesized in the water also presents non-magnetic results. SEM results shown in Figure S2 (a-e) indicate the particle size of γ-Fe_2_O_3_ is increased with the improvement of heating rates. The sample synthesized in water shown a bulk and dispersive α-Fe_2_O_3_ particles.


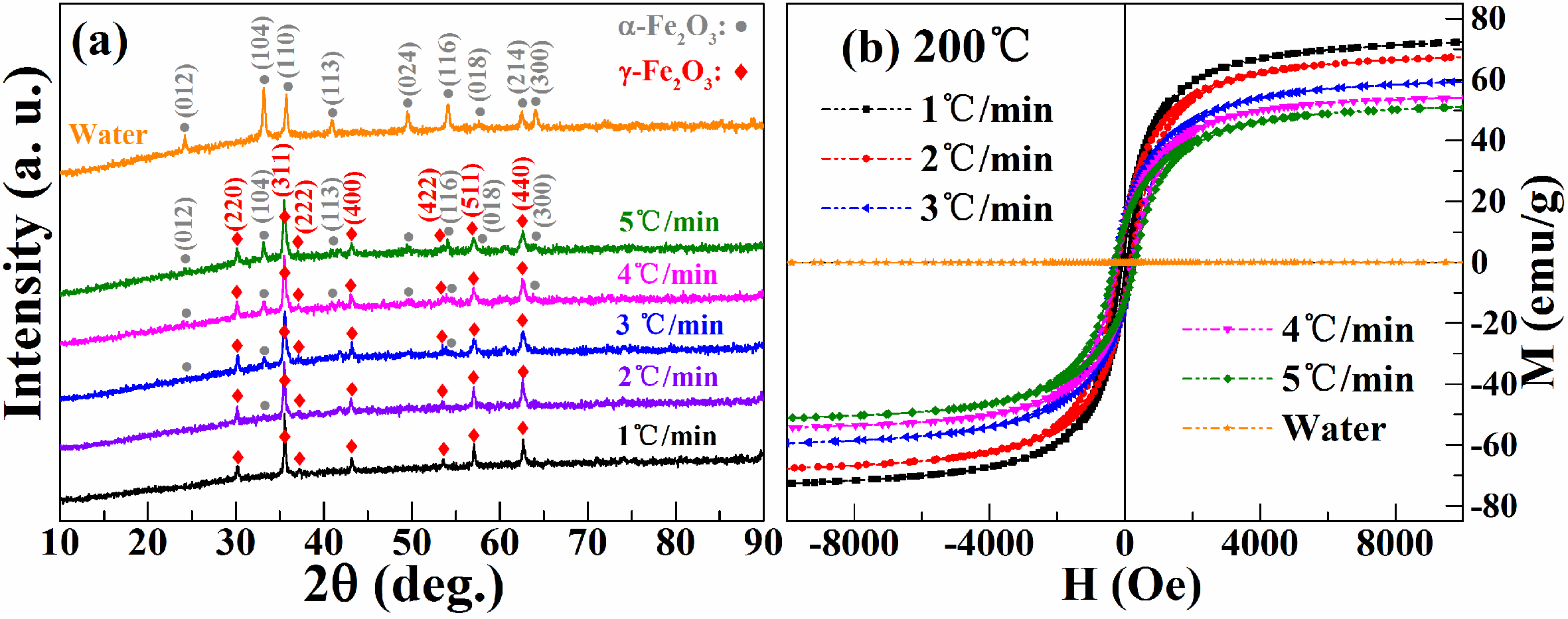


**Figure S1.** Heating rates dependence of XRD patterns (a) and VSM results (b) with 200^o^C for 2 hours. The sample named "water" in (a) and (b) is the results synthesized at 200^o^C for 2 hours, and its heating rate was 1^o^C/min.


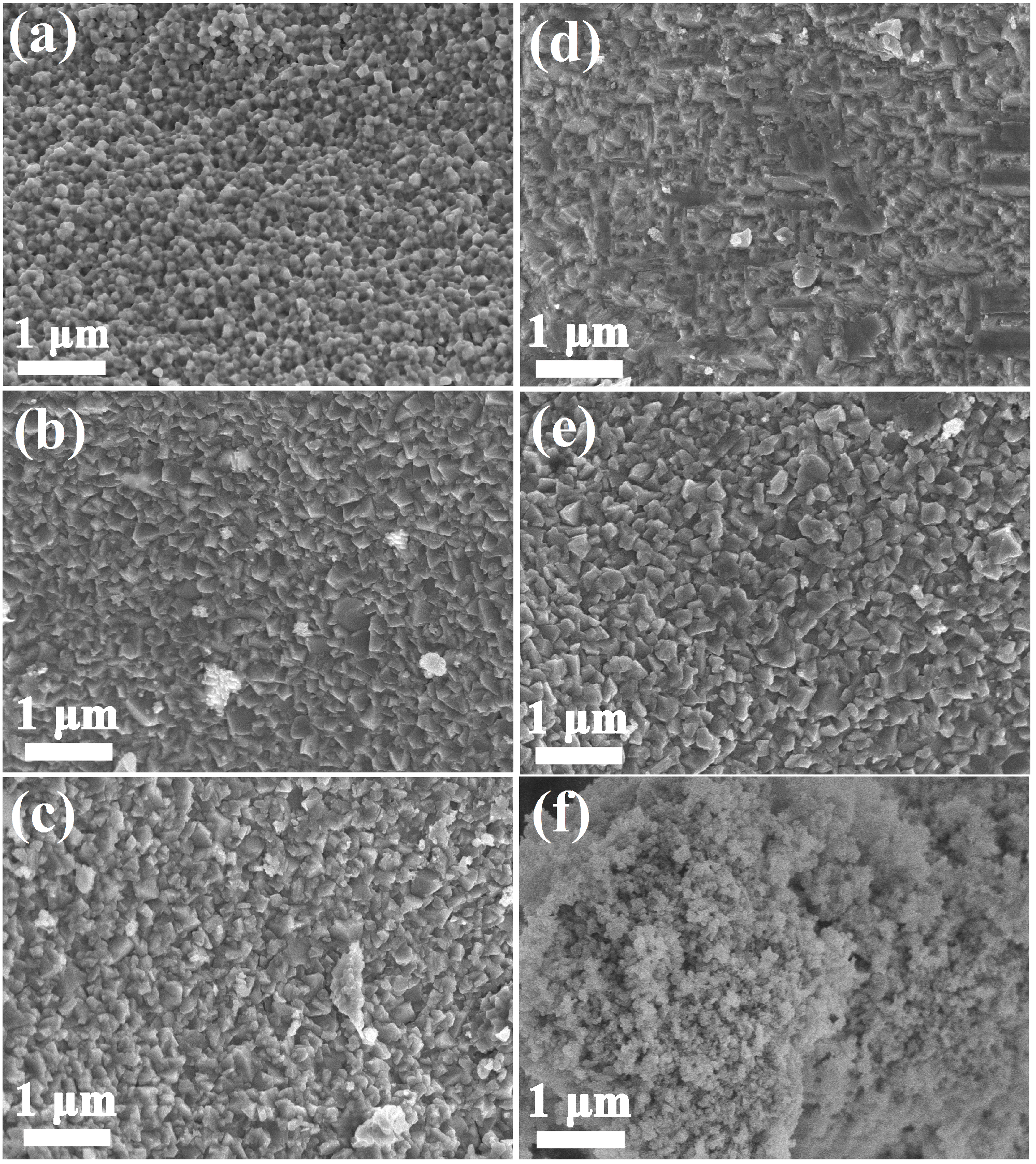


**Figure S2.** Heating rates dependence of SEM results (a-e): 1^o^C/min, 2^o^C/min, 3^o^C/min, 4^o^C/min, and 5^o^C/min respectively; picture (f) is synthesized in water.

Figure S3 (a) demonstrates the evolution of MB absorption spectra at different irradiation times, from which we can see that the concentration of MB decreased ordinarily. Figure S3 (b) shows the photocatalytic degradation ability of MB of *γ*-Fe_2_O_3_ after the sample suspension was stirred for 40 min in the dark. The result indicates that *γ*-Fe_2_O_3_ has little adsorption ability of MB. Additionally, the *γ*-Fe_2_O_3_ shows proper photocatalytic activity, which can degrade 16% MB dye in 60 min under UV irradiation. The degradation of MB can be ascribed to a pseudo-first-order reaction with a Langmuir–Hinshelwood model when C_0_ is small[^1^](#_ENREF_1):

*ln*(*C_0_*/*C*)=*kt*

where *k* is the apparent first-order rate constant, as displayed in Figure S3 (b) of inset. Higher slope indicates the faster rate of degradation for the *γ*-Fe_2_O_3_ photocatalyst. Fairly good linear plots can be observed, indicating that all the reactions followed the first-order kinetics, and the photocatalytic activity of this work is comparable with the previous work[^2^](#_ENREF_2). Figure S3 (c-d) shows the nitrogen adsorption−desorption isotherms and corresponding Barrett-Joyner-Halenda (BJH) pore size distribution plots of *γ*-Fe_2_O_3_ nano-particles at 200^o^C.


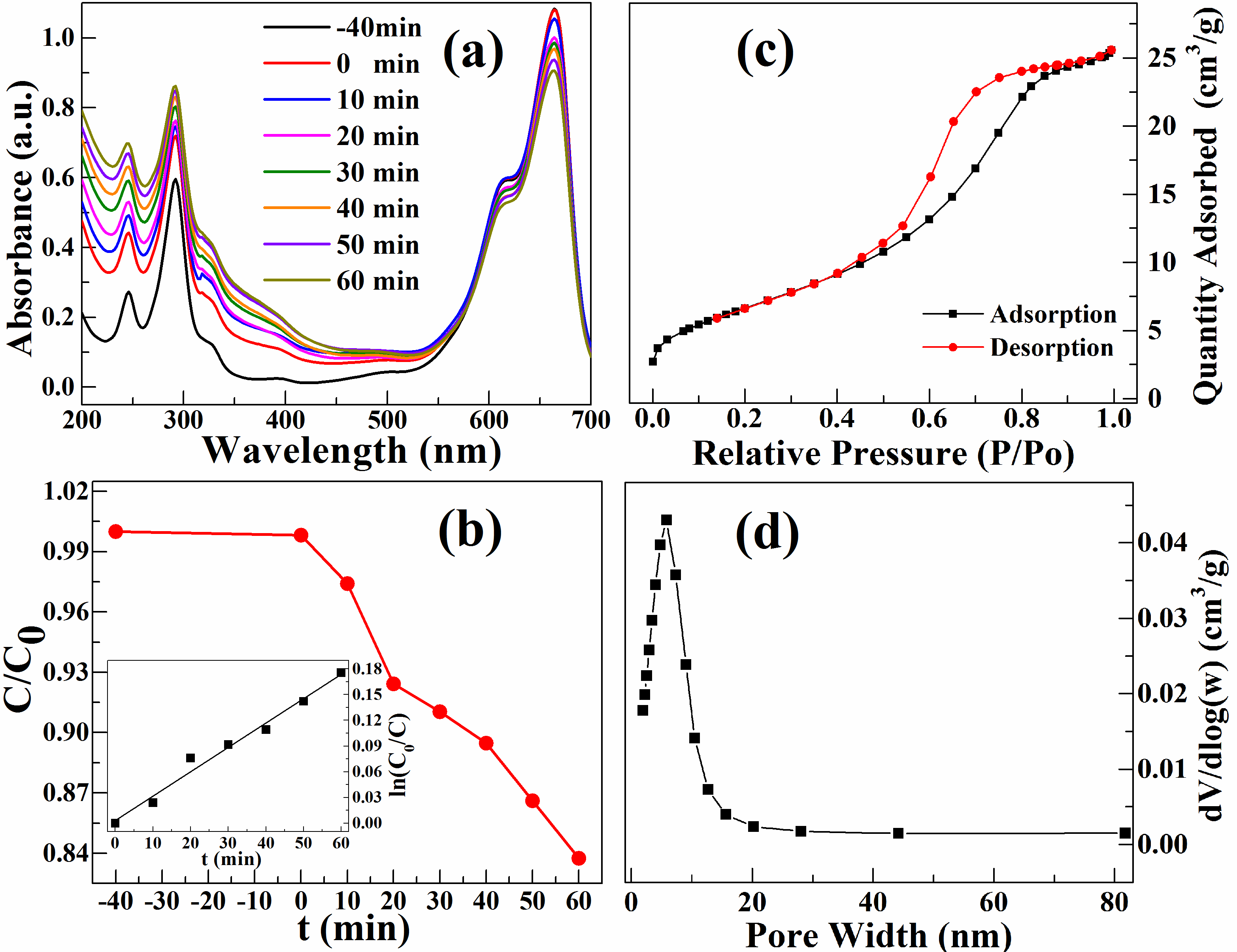


**Figure S3.** Diffuse reflectance ultraviolet-visible absorption spectra of MB solutions withdrawn at selected times (a); Photocatalytic degradation ratio of MB solution in the presence of *γ*-Fe_2_O_3_, and the inset shows the Plots of ln (C_0_/C) versus irradiation time (b); Nitrogen adsorption−desorption isotherms (c) and BJH pore size distribution curves (d).

**References**

1 Wang, X. H., Li, J. G., Kamiyama, H., Moriyoshi, Y. & Ishigaki, T. Wavelength-Sensitive Photocatalytic Degradation of Methyl Orange in Aqueous Suspension over Iron(III)-doped TiO_2_ Nanopowders under UV and Visible Light Irradiation. *J. Phys. Chem. B* **110**, 6804-6809 (2006).

2 Chen, F. & Zhao, J. Preparation and photocatalytic properties of a novel kind of loaded photocatalyst of TiO_2_/SiO_2_/γ‐Fe_2_O_3_. *Catal. Lett.* **58**, 246-247 (1999).

3 Xu, J.-S. & Zhu, Y.-J. Monodisperse Fe_3_O_4_ and γ-Fe_2_O_3_ magnetic mesoporous microspheres as anode materials for lithium-ion batteries. *ACS Appl. Mater. Interfaces* **4**, 4752-4757 (2012).

4 Asuha, S., Zhao, Y. M., Zhao, S. & Deligeer, W. Synthesis of mesoporous maghemite with high surface area and its adsorptive properties. *Solid State Sci.* **14**, 833-839 (2012).

5 Ianos, R., Taculescu, E.-A., Pacurariu, C. & Niznansky, D. gamma-Fe_2_O_3_ nanoparticles prepared by combustion synthesis, followed by chemical oxidation of residual carbon with H_2_O_2_. *Mater. Chem. Phys.* **148**, 705-711 (2014).

1. ^*^ Corresponding author: liuqf@lzu.edu.cn (Qingfang Liu)

   Tel: +86-931-8914171, Fax: +86-931-8914160 [↑](#footnote-ref-2)
